# State of Cancer Control in Rwanda: Past, Present, and Future Opportunities

**DOI:** 10.1200/GO.20.00281

**Published:** 2020-07-23

**Authors:** Fidel Rubagumya, Ainhoa Costas-Chavarri, Achille Manirakiza, Gad Murenzi, Francois Uwinkindi, Christian Ntizimira, Ivan Rukundo, Pacifique Mugenzi, Belson Rugwizangoga, Cyprien Shyirambere, Sandra Urusaro, Lydia Pace, Lori Buswell, Faustin Ntirenganya, Emmanuel Rudakemwa, Temidayo Fadelu, Tharcisse Mpunga, Lawrence N. Shulman, Christopher M. Booth

**Affiliations:** ^1^ Department of Oncology, Rwanda Military Hospital, Kigali, Rwanda; ^2^ University of Global Health Equity, Burera, Rwanda; ^3^ Department of Surgery, Rwanda Military Hospital, Kigali, Rwanda; ^4^ Department of Research, Rwanda Military Hospital, Kigali, Rwanda; ^5^ Rwanda Biomedical Center, Kigali, Rwanda; ^6^ City Cancer Challenge, Kigali, Rwanda; ^7^ Department of Radiology, Rwanda Military Hospital, Kigali, Rwanda; ^8^ Department of Pathology, Kigali University Teaching Hospital, Kigali, Rwanda; ^9^ Department of Surgery, Kigali University Teaching Hospital, Kigali, Rwanda; ^10^ Department of Radiology, King Faisal Hospital, Kigali, Rwanda; ^11^ Department of Oncology, Inshuti Mu Buzima, Kigali, Rwanda; ^12^ Division of Women’s Health, Brigham and Women’s Hospital, Boston, MA; ^13^ Department of Oncology, Dana-Farber Cancer Institute, Boston, MA; ^14^ Rwanda Ministry of Health, Kigali, Rwanda; ^15^ Center for Global Cancer Medicine, University of Pennsylvania, Philadelphia, PA; ^16^ Department of Oncology, Queen’s University, Kingston, ON, Canada

## Abstract

Rwanda is a densely populated low-income country in East Africa. Previously considered a failed state after the genocide against the Tutsi in 1994, Rwanda has seen remarkable growth over the past 2 decades. Health care in Rwanda is predominantly delivered through public hospitals and is emerging in the private sector. More than 80% of patients are covered by community-based health insurance (Mutuelle de Santé). The cancer unit at the Rwanda Biomedical Center (a branch of the Ministry of Health) is responsible for setting and implementing cancer care policy. Rwanda has made progress with human papillomavirus (HPV) and hepatitis B vaccination. Recently, the cancer unit at the Rwanda Biomedical Center launched the country’s 5-year National Cancer Control Plan. Over the past decade, patients with cancer have been able to receive chemotherapy at Butaro Cancer Center, and recently, the Rwanda Cancer Center was launched with 2 linear accelerator radiotherapy machines, which greatly reduced the number of referrals for treatment abroad. Palliative care services are increasing in Rwanda. A cancer registry has now been strengthened, and more clinicians are becoming active in cancer research. Despite these advances, there is still substantial work to be done and there are many outstanding challenges, including the need to build capacity in cancer awareness among the general population (and shift toward earlier diagnosis), cancer care workforce (more in-country training programs are needed), and research.

## INTRODUCTION

Cancer is recognized as a global problem. Recent reports indicate that 1 in 5 men and 1 in 6 women worldwide will develop cancer in their lifetime.^1^ In 2018, 18.1 million new cases were reported worldwide, with many of them occurring in low- and middle-income countries. Approximately 1 million new cases are diagnosed annually in Africa.^2^ The etiology behind the rising cancer burden in low- and middle-income countries, and Rwanda in particular, is likely to be multifactorial and includes changes in demographics and lifestyle.

**CONTEXT**

**Key Objective**
To describe the current state of cancer control in Rwanda, challenges faced over the years, and future strategic plans.
**Knowledge Generated**
Over the past 2 decades, Rwanda has invested resources in building a vibrant health care sector that includes oncology. Cancer control gained momentum in 2012 with the establishment of the Butaro Cancer Center of Excellence. Since then, much has been achieved in terms of cancer policy, advocacy, screening, diagnosis, and treatment.
**Relevance**
Rwanda plans to become a health care hub and to develop medical tourism (where people travel to obtain medical treatment) by attracting state-of-the-art and specialized medical facilities. This cannot be achieved without well-functioning cancer care.

In this article, we describe the current status of can-cer care and control in Rwanda. We review different aspects of cancer control as outlined by the WHO, including cancer policy and advocacy; cancer prevention, screening, and early detection; cancer diagnosis and treatment; palliative care in oncology; and human resources for oncology. Our purpose is to provide an overview of cancer care in Rwanda and to highlight system-level issues that require attention over the next 5 years (Table 1).

**TABLE 1:**
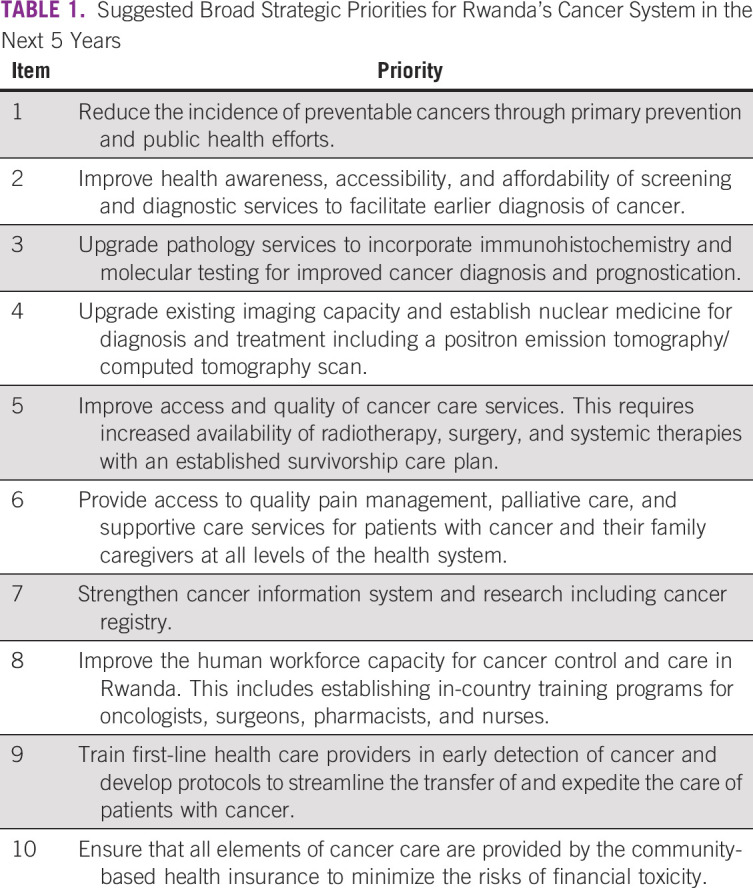
Suggested Broad Strategic Priorities for Rwanda’s Cancer System in the Next 5 Years

## HEALTH CARE DELIVERY IN RWANDA

Located in East Africa, Rwanda is a landlocked country with a population of 12.1 million. Rwanda is a low-income country with a gross domestic product of US$9.1 billion (2019) and an estimated annual expenditure on health care per capita of US$53.^3,3a^ Current life expectancy is 67 years.^4^ Considered a failed state after the genocide against the Tutsi in 1994, Rwanda has experienced remarkable growth over the past 2 decades. Substantial investment has been made in health care, infrastructure, and education.^3^

Health care in Rwanda is predominantly delivered through public hospitals and is emerging in the private sector. The health care sector is organized around tertiary university teaching hospitals, provincial referral hospitals, district hospitals, health centers, and health posts (the newest type of health care facility). Patients are referred bottom to top, from a health post to a university teaching hospital, and more than 80% of patients are covered by community-based health insurance (Mutuelle de Santé).^5,6^ Precise data on cancer incidence and mortality is unavailable because a cancer-specific population-based registry was resumed only in 2018. However, GLOBOCAN 2018 estimated a total of 10,704 cancer cases and 7,662 deaths.^7^ Cancer incidences are expected to increase globally, and with that increase comes the inevitable need to consider cancer as a significant and growing public health concern that requires attention, investment, and prioritization by the government and by nongovernmental and international organizations working in Rwanda.

## CANCER POLICY AND ADVOCACY

The Rwanda Biomedical Center of the Ministry of Health (RBC/MOH) is the nation’s central health implementation agency. The Non-Communicable Diseases (NCD) Division of the RBC oversees all NCD activities. Housed within the NCD Division is a cancer unit with a dedicated team that sets and implements cancer policy. A National Cancer Control Plan (NCCP), based on WHO guidelines, was launched on World Cancer Day in February 2020.^8^ National cancer disease-specific guidelines are being written by a collaborative, multidisciplinary group of health care professionals with oversight provided by the RBC. There are now several advocacy groups, such as Rwanda Children’s Cancer Relief , Breast Cancer Initiative East Africa, and Conquer Breast Cancer. These groups have helped raise public awareness of cancer, promote early detection programs, and provide psychosocial support to patients with cancer and survivors.^9,10^

## CANCER PREVENTION, EARLY DETECTION, AND SCREENING

There is still a large gap in cancer prevention, early detection, and screening in Rwanda. However enormous strides have been made in some areas such as providing human papillomavirus (HPV) vaccination for girls age 9 and 12 years, encouraging physical activity (car-free days), and fighting HIV/AIDS.^11-13^ The country’s HPV vaccination program achieved 80% to 90% for children born from 2001 to 2006 (children age 12 years by the time of vaccination).^14^ Other cancer prevention measures such as infection control (vaccination for hepatitis B virus and treatment for hepatitis C virus), smoking cessation, and curbing air pollution are now standard public health issues. There is an urgent need to improve health awareness, which would facilitate early diagnosis. A recent study found that 52% of patients with breast cancer in Rwanda present with stage III and 24% present with stage IV; this is a result of both system- and patient-related delays in having cancer diagnosed.^15^

The recently launched NCCP indicates some of the cancers for which screening and early detection are recommended: cervical, breast, and colorectal cancers. Rwanda has followed WHO recommendations for screen and treat strategies for cervical cancer and performs screening by using HPV testing and triaging with visual inspection with acetic acid (VIA) before treatment or by using VIA and treatment where HPV testing is not available.^16^ Rwanda’s national cervical cancer screening program is currently halfway implemented; 50% of health facilities now have trained health care providers and functional women’s cancer clinics (WCCs). These clinics combine cervical and breast cancer screening and early detection, and the nation’s target is to have WCCs at all health facilities nationwide.^17^

Breast cancer awareness has increased significantly through public campaigns and mobilization of breast cancer survivor networks. Efforts to improve early detection of breast cancer have focused on educating community health care workers in performing clinical breast examinations and using appropriate referral protocols.^18^ These training programs are being expanded across the country. Further improvements in early detection of breast cancer will include reducing the delay from presentation to diagnosis by expanding breast imaging capabilities for symptomatic and high-risk individuals and increasing the number health care professionals trained to recognize early signs and symptoms of the disease.

## CANCER DIAGNOSTICS, TREATMENT, AND PALLIATIVE CARE

### Radiology

Medical imaging is a critical element of cancer care systems.^19^ In Rwanda, medical imaging has developed over time and plays an integral role in the screening and early detection, diagnosis, staging, and follow-up of cancer, which ensures the continuum of cancer management. Most public and private hospitals have conventional and/or digital radiography facilities, ultrasound machines are available in all district hospitals, but only 5 institutions have mammography services.^20^ Computed tomography (CT) scans are available only at University Teaching Hospitals and 2 national referral hospitals. King Faisal Hospital (KFH) is the only hospital in the public health system that can offer 1.5T magnetic resonance imaging (MRI). There is also a 1.5T MRI system in private practice. Positron emission tomography/CT (PET/CT) and PET/MRI are not yet available in Rwanda. At this time, some interventional radiology procedures (ie, ultrasound-guided fine needle aspiration biopsies, drains, and CT-guided biopsies) are available only in private hospitals. Imaging capacity has increased, especially in private hospitals and clinics in Kigali, which has helped reduce waiting times. However, Rwanda’s overall capacity is still below what is needed.

### Pathology Services

Pathology is another essential element of cancer care delivery. The history of anatomic pathology in Rwanda is an integral chapter in Rwanda’s history of medicine. Frans Vanderick, MD, the founding Dean of the Faculty of Medicine at the National University of Rwanda, started the anatomic pathology service when the medical school was founded in 1963.^21^ Until 2009, this was the only pathology service in the country. Between 2010 and 2015, 8 Rwandan physicians were sent abroad for training and who returned to clinical practice. Before 2013, pathology services in Rwanda had been delivered by expatriates who, among many other achievements, had started 3 more pathology laboratories: KFH Kigali (KFH-K) in 2009, Butaro Cancer Center of Excellence (BCCOE) in 2012, and the University Teaching Hospital of Kigali in November 2013, which is the largest hospital in Rwanda. Rwanda Military Hospital started a pathology service in 2015, and some private pathology services began at the same time. Anatomic pathology services are still limited to routine histopathology and cytopathology, histochemistry, and immunohistochemistry; the latter remains a challenge because of the lack of several reagents. Flow cytometry and some molecular pathology techniques are not available for routine clinical use. In addition to training pathologists, some laboratory scientists were also sent abroad for hands-on training in advanced diagnostic techniques, and others were trained locally by visiting experts from the American Society of Clinical Pathologists. At BCCOE, telepathology allows for expert consultation with colleagues from cancer centers in other countries.

The Government of Rwanda approved the launch of a program for pathology postgraduate residency at what was then known as the National University of Rwanda in April 2013.^22^ As of November 2019, 9 physicians have completed their local training as pathologists with the support of US centers.^23^ The quality of pathology services in Rwanda has since greatly improved. Although the number of cancer cases reported was less than 300 per year before 2004,^24^ it exceeded 3,000 in 2018.^25^ But the detection rate is likely still low, because the WHO estimates that about 10,000 new cancer cases occur in Rwanda each year.^26^ Thus, plans have been developed for improving pathology services in Rwanda by investing in workforce and infrastructure.^27^

### Treatment of Cancer

Before 2012, Rwanda’s health priorities were focused on curbing infectious diseases and maternal mortality.^28,29^ Since then, the Government of Rwanda has made great strides in providing health care to patients with cancer.

#### Chemotherapy.

Initial efforts came from the BCCOE, which was established in 2012 through collaboration with Partners in Health (PIH). Before then, cancer care was limited to surgery and supportive care.^29,30^ With assistance from the Dana-Farber Cancer Institute, BCCOE began by focusing its efforts on highly treatable malignancies and scaled up over time, using cancer chemotherapy agents found on the WHO Essential Medicine List.

Because of the lack of trained medical oncologists, cancer treatment at BCCOE (including chemotherapy) is provided by physicians who are not oncologists. These providers have received cancer-specific training, and they follow treatment guidelines developed at BCCOE.^31^ Oncologists from the United States and Canada provide support on weekly tumor board conference calls.^31^ In addition, annual visits by specialist oncologists, pathologists, and surgeons provide clinical and educational support and advice on systems of care. As capacity grows, the center will become increasingly staffed by oncologists trained in Rwanda.^31^

Although the government has plans to expand services, chemotherapy is currently available only in the public sector at BCCOE and in the private sector at KFH. This is largely a result of the challenges of financing cancer care. Most cytotoxic drugs are available in select retail pharmacies; however, they are not covered by community-based health insurance. At BCCOE, drugs are provided free of charge to patients, an effort supported by financing from the nongovernmental PIH organization. Targeted drugs such as trastuzumab and rituximab and immunotherapies such as nivolumab and pembrolizumab are expensive enough to be unaffordable and out of reach for most Rwandans. The one exception is imatinib for treating patients with chronic myeloid leukemia or gastrointestinal stromal tumors; it is provided free of charge by the Max Foundation and the Glivec Access Patient Assistance Program.

Skilled and knowledgeable oncology nurses are required to safely deliver chemotherapy and care for patients with cancer. In a collaborative effort, BCCOE, PIH, and Dana-Farber Cancer Institute developed a twinning model for oncology nurses that has been in effect since 2012. This model places a US-based oncology nurse on site for 3 to 15 months to serve as a mentor. This model has resulted in a 3-week training program for all nurses new to the BCCOE, provision of oncology-specific competency check lists, policies, and procedures, refresher training for nurses, and most importantly, 2 Rwandan oncology nurse educators to carry out training and provide leadership and advocacy for nurses and patients.^32^ The Government of Rwanda has also launched a formal master’s degree program in nursing at the University of Rwanda School of Nursing and Midwifery, with oncology as 1 of the 8 tracks to ensure a pipeline of oncology nurses for the future. Nurses fill a multitude of roles within the specialty of oncology as educators, researchers, navigators, care providers across the continuum, leaders, and advocates. Rwanda is well positioned to scale up, expand over time, and tap into this nursing potential to serve the residents of Rwanda and neighboring countries.

#### Surgery.

Patients with cancer who need surgery are managed mostly at tertiary hospitals in the City of Kigali, and some are cared for at the BCCOE. However, data on outcomes are limited. Currently, there are few residency training programs for general specialists in orthopedics, neurosurgery, and urology. Because there are no surgical oncology fellowships in Rwanda, surgeries are performed by general specialists who may not have training in surgical oncology. Complex resections and procedures (such as those for liver and pancreatic cancers) are therefore not possible. Plans are underway to develop a breast surgery fellowship at Rwanda Military Hospital.

#### Radiation.

For most of the current decade, radiation therapy has not been available in Rwanda. A small number of patients who need radiotherapy enrolled under the PIH program and had to be referred to surgical centers abroad for treatment.^33,34^ The first radiation therapy center in Rwanda started treating patients at Rwanda Military Hospital in early 2019. Since then, more than 500 new patients have been treated. The center has 2 linear accelerators with volumetric modulated arc therapy capacity, and there are plans to add 1 high-dose-rate brachytherapy machine. The center provides radiotherapy services for all Rwandans and also accepts patients from underserved neighboring countries. The waiting time for both curative and palliative radiotherapy currently ranges from 1 to 4 weeks. The waiting time is reasonably short, mainly because radiotherapy is a new cancer service in the country, and most patients, especially those who need palliative radiotherapy, are not referred.

Expanded availability of surgery, chemotherapy, and radiation therapy services has substantially reduced the number of referrals to treatment facilities outside the country. However, affordability of cancer care remains a major challenge. Despite community-based health insurance that covers 90% of non-chemotherapy medical bills, the remaining 10% is still a major barrier for many patients who are diagnosed with cancer. This is mainly because of numerous hospital visits for chemotherapy infusion, follow-up visits, and complementary investigations, which lead to significant financial toxicity.

### Palliative and Supportive Care

Rwanda was the first African country to adopt a stand-alone national policy on palliative care, as well as strategic and implementation plans for palliative care.^35^ With the support of international partners, including Intrahealth and Mildmay, the MOH conducted a pilot program of integrating palliative care into the existing health system. This was accomplished by providing education and training in palliative care and especially in pain assessment and management.^36^ As in many places, it was evident that the main challenge in the field of palliative care was the fear of prescribing morphine because of the myths surrounding its adverse effects despite the existing Single Convention on Narcotic Drugs.^37^ As a result, in 2012, the MOH conducted trainings in pain assessment and management for palliative care, which in turn changed physicians’ attitudes toward treating pain.^38^

Between 2007 and 2009, the Rwanda MOH reported annual opioid prescription of approximately 0.2 kg, which could treat only 27 people at that time. In addition, a report from the Lancet Commission on Global Access in Pain Control and Palliative care revealed an abyss between developing and developed countries.^39^ According to the RBC, national opioid consumption increased from 0.2 kg to 9 kg in 2018. The Government of Rwanda started to convert its morphine from powder to syrup (5 mg of morphine per 5 mL of syrup) at Huye District, which is free to all patients with cancer under the palliative care program. Law N* 03/2015 or 15/02/2012, Article 17, which governs narcotic drugs, psychotropic substances, and precursors in Rwanda authorized dentists, veterinarians, qualified midwives, or nurses to prescribe narcotic drugs and psychotropic substances.^40^ In collaboration with civil societies, the College of Medicine and Health Sciences at the University of Rwanda is developing a curriculum in palliative care, an important step in multidisciplinary task-shifting in which future medical doctors, nurses, and midwives will work together with a focus on inpatient-centered services.

Despite the progress made with the development of palliative care policy and community-based services,^41^ health care providers still consider palliative care as a fifth wheel in cancer care. Patients are primarily referred for terminal care, and there is still fear of prescribing opioids, which is why there is still a low level of opioid use in Rwanda. The integration of palliative care into the Rwanda NCCP is an important step toward securing dignity and humanity for all patients with cancer.

## CANCER RESEARCH

Cancer research in Rwanda initially came from the original epidemiologic cancer registry in the southern prefecture of Butare.^42,43^ This hospital cancer registry was originally in place at the University Teaching Hospital of Butare from 1991 to 1994; it resumed its activities from 2010 to 2014 and was funded by the US National Institutes of Health through collaborative efforts of the Rwanda Military Hospital and the Albert Einstein College of Medicine; about 6,000 patients were registered, mainly in hospitals in Kigali. In 2018, the government of Rwanda through the MOH/RBC started a state-owned national cancer registry based on previous efforts by the Rwanda-Einstein collaborations.

Cancer research in Rwanda has mostly consisted of limited efforts at the different hospitals involved in cancer care, especially BCCOE, University Teaching Hospital of Kigali, and Rwanda Military Hospital. Most of the research output has focused on epidemiology, cancer care delivery models with minimal output in oncology basic science, translational studies, and interventional clinical trials. BCCOE has been exceptional at producing numerous implementation sciences and randomized interventional studies. Cancer research has been hampered by lack of trained human resources, lack of funding opportunities, and lack of incentives for clinicians to be involved in cancer research. Ongoing efforts by the government to develop national cancer protocols and to improve cancer care coordination across the various hospitals has led to increased collaboration. Future plans for cross-site research projects are being discussed, especially in the areas of breast and GI cancers. A national cancer symposium was launched in February 2020 on World Cancer Day. Planned as an annual event, it will serve as a platform for sharing cancer research output in the country.

## HUMAN RESOURCES FOR CANCER CARE

Rwanda still has limited human resources for cancer care. At this time as per Rwanda Medical and Dental Counsel, there are few formally trained oncologists: 5 clinical oncologists, 1 pediatric oncologist, no surgical oncologists, no gynecology or medical oncologists, and 19 nurse oncologists. There are 15 general pathologists and 15 general radiologists. Trainees in different oncology and related subspecialties both in and out of the country will eventually augment current numbers. Training programs in pathology and radiology have already been approved. Because it will take some time to have in-country training programs for clinical oncology and surgical oncology, Rwandan centers have become creative with task-sharing. This has been the case for BCCOE since its inception along with several other training opportunities for surgeons.^29^ In addition to oncologists, support staff such as onco-pharmacists, pharmacy technicians, oncology nurses, radiation therapy technicians, medical physicists, and palliative care specialists are also needed.^44^

## CHALLENGES AND NEXT STEPS

The substantial work that still needs to be done has many outstanding challenges and needs. Some of the challenges include the need to increase cancer awareness among the general population (and shift toward earlier diagnosis), training more oncologists in all fields (nursing and surgical, medical and radiation oncology) and other allied professionals in cancer care. In addition, it is vital to ensure a consistent supply of all cancer drugs, especially those on the WHO Essential Medicine List, and especially those needed for palliative care. Because of the extreme financial toxicities caused by out-of-pocket expenses, cancer care (from screening, diagnosis, and treatment to follow-up) should be fully covered by the community-based insurance scheme (Mutuelle de Santé). Rwanda needs to strengthen and maintain the national cancer registry, put in place funding mechanisms for cancer research, and incentivize clinicians by providing training opportunities to do more cancer research. Finally, strengthening and integrating palliative care within oncology services is a pressing priority.

In conclusion, Rwanda has made tremendous progress in cancer control over the last few years. Progress is evident by looking at a country that in the past could not provide any form of cancer treatment to its currently housing 2 functional cancer centers. In addition, there are several tertiary hospitals that provide cancer care and a national cancer unit within the MOH. The past decade has been a period of exciting changes within Rwanda’s cancer care system. Further investment and ongoing collaboration between in-country and out-of-country clinicians, researchers, and policy makers will remain essential as we work toward having accessible and high-quality care available for all Rwandans with cancer.
